# Drinking patterns, craving and readiness to change among Hungarian highly educated women – Findings from three pilot studies

**DOI:** 10.1192/j.eurpsy.2026.12042

**Published:** 2026-07-31

**Authors:** S. Gubucz-Pálfalvi, T. Kurimay, I. Danis

**Affiliations:** 1 Mental Health Sciences Division, Interdisciplinary Social Sciences Program, Doctoral College of Semmelweis University; 2 Family-Centred Mental Health Centre, North Buda Saint John’s Central Hospital; 3 Faculty of Health and Public Services, Institute of Mental Health, Semmelweis University, Budapest, Hungary

## Abstract

**Introduction:**

Alcohol consumption among women has been steadily increasing in recent decades, narrowing the gender gap in hazardous and harmful alcohol consumption.Despite this trend, little is known about the psychosocial and behavioural characteristics of alcohol use, especially among highly educated women. Understanding these characteristics is crucial for developing effective prevention and intervention strategies for this special target population.

**Objectives:**

This study aimed to examine alcohol use patterns, craving, emotional well-being, and readiness to change in highly educated women who responded in three online pilot studies in Hungary.

**Methods:**

Between 2023 and 2025, three online validation pilot studies were conducted in Hungary, using convenience and snowball sampling, as preparatory work for a forthcoming nationally representative survey. These studies provided a unique opportunity to investigate a rarely examined population, as the samples were strongly enriched with highly educated women. After data cleaning, a total of 851 responses were retained — all from women with a university degree — resulting in a uniquely homogenized and highly qualified sample. Data were collected online via LimeSurvey. The survey package included the Alcohol Use Disorders Identification Test (AUDIT), the Hospital Anxiety and Depression Scale (HADS), the PENN Alcohol Craving Scale (PACS), and the Stages of Change Readiness and Treatment Eagerness Scale (SOCRATES), along with sociodemographic questions, tobacco and drug use, and psychotropic medication use.

**Results:**

Preliminary analyses of a homogeneous sample of 851 highly educated women from Hungary showed a skewed distribution of AUDIT scores toward lower risk, with relatively few participants scoring in the higher-risk range. Using the traditional AUDIT cut-off (≥ 8), appr. 20% of participants met criteria for hazardous or harmful alcohol use. Applying the WHO female-specific cut-off (≥ 5), the proportion of hazardous drinkers rose to appr. 40%. Additionally, appr. 8% scored ≥ 15, indicating probable alcohol dependence.

Further analyses and results — including scale descriptives, subgroup comparisons by age and other sociodemographic characteristics as well as the associations between craving, readiness to change, and other measured constructs —will be discussed in detail during the presentation.

**Conclusions:**

The prevalence of hazardous drinking observed in this homogeneous sample of highly educated Hungarian women appears to be higher than what is typically reported for the general female population. These pilot studies offer new insights into alcohol use behaviour, craving, and psychological correlates in this special population. Further analyses will examine the underlying factors behind this elevated risk.

The findings may inform the development of tailored prevention and intervention strategies for similar populations.

**Disclosure of Interest:**

None Declared

